# Monitoring Acclimatization and Training Responses Over 17–21 Days at 1,800 m in Elite Cross-Country Skiers and Biathletes

**DOI:** 10.3389/fspor.2022.852108

**Published:** 2022-05-11

**Authors:** Øyvind Karlsson, Marko S. Laaksonen, Kerry McGawley

**Affiliations:** Swedish Winter Sports Research Centre, Department of Health Sciences, Mid Sweden University, Östersund, Sweden

**Keywords:** altitude, athlete monitoring, endurance sports, hypoxia, Nordic skiing, winter sports

## Abstract

**Objective:**

To monitor the daily variations and time course of changes in selected variables during a 17–21-day altitude training camp at 1,800 m in a group of elite cross-country skiers (9 women, 12 men) and biathletes (7 women, 4 men).

**Methods:**

Among other variables, resting peripheral oxygen saturation (SpO_2rest_), resting heart rate (HR_rest_) and urine specific gravity (USG) were monitored daily at altitude, while illness symptoms were monitored weekly. Before and after the camp, body composition (i.e., lean and fat mass) and body mass were assessed in all athletes, while roller-skiing speed at a blood lactate concentration of 4 mmol·L^−1^ (Speed_@4mmol_) was assessed in the biathletes only.

**Results:**

Neither SpO_2rest_, HR_rest_ nor USG changed systematically during the camp (*p* > 0.05), although some daily time points differed from day one for the latter two variables (*p* < 0.05). In addition, body composition and body mass were unchanged from before to after the camp (*p* > 0.05). Eleven out of 15 illness episodes were reported within 4 days of the outbound or homebound flight. The five biathletes who remained free of illness increased their Speed_@4mmol_ by ~ 4% from before to after the camp (*p* = 0.031).

**Conclusions:**

The present results show that measures typically recommended to monitor acclimatization and responses to altitude in athletes (e.g., SpO_2rest_ and HR_rest_) did not change systematically over time. Further research is needed to explore the utility of these and other measures in elite endurance athletes at altitudes typical of competition environments.

## Introduction

It is common to incorporate altitude exposure into elite endurance athletes' training schedules to induce short- and long-term physiological adaptations that may improve performance at altitude and/or sea level (Millet et al., 2010; Burtscher et al., 2018a; Mujika et al., 2019). Altitude training is typically included in the annual training plan as 2–4-week camps at low to moderate altitudes (i.e., ~ 1,400–2,500 m) (Millet et al., 2010; Mujika et al., 2019). While there are conflicting views on the effects of altitude training on subsequent sea-level performance (Lundby and Robach, 2016; Millet et al., 2019; Millet and Brocherie, 2020; Siebenmann and Dempsey, 2020), there is consensus that acclimatization to altitude is necessary for optimal performance at altitude (Chapman et al., 2013; Burtscher et al., 2018a). Current recommendations suggest that ~ 14 days of acclimatization at the same altitude as the competition is sufficient at moderate to high altitudes (i.e., 2,000–4,500 m) (Bärtsch et al., 2008; Bergeron et al., 2012; Chapman et al., 2013; Burtscher et al., 2018a). However, since the time required for acclimatization is likely to be altitude dependent, less time may be sufficient to acclimatize to lower altitudes (Bärtsch et al., 2008; Bergeron et al., 2012; Chapman et al., 2013; Burtscher et al., 2018a). Despite this, very little systematic research attention has been given to acclimatization at low altitudes (i.e., <2,000 m) in elite endurance athletes (Burtscher et al., 2018a). In cross-country (XC) skiing and biathlon, international competitions are limited to a maximal altitude of 1,800 m (International Biathlon Union, 2020; International Ski Federation, 2020) (e.g., the XC skiing World Cup in Davos, ~ 1,560 m; the 2020 Biathlon World Championships in Antholtz, ~ 1,600 m and the 2022 Beijing Olympics events were held at ~ 1,700 m). As such, training camps based at altitudes <2,000 m are regularly incorporated into these athletes' training plans. From an applied research perspective, it seems pertinent to understand athlete responses on ascent to and during sojourns at these altitudes.

Whether altitude training is employed to improve subsequent sea-level performance or to prepare for competition at altitude, a main goal is to maximize the positive physiological adaptations. Careful monitoring of individual responses to training loads (TLs) and acclimatization is essential for optimizing these adaptations and for reducing the risk of adverse effects, such as over-training, illness and/or dehydration (Saw et al., 2016; Mujika et al., 2019; Stellingwerff et al., 2019). Given the combined stress of hypoxia and potential increases in TLs, individual monitoring is especially important during altitude training camps where large inter-individual differences in responses to hypoxia may manifest (Chapman, 2013). While multiple indices for monitoring TLs, training responses and acclimatization have been suggested (Saw et al., 2016; Sperlich et al., 2016; Mujika et al., 2019), the invasive and/or time-consuming nature of measuring blood markers and implementing multi-item questionnaires, for example, makes their daily use with a large group of elite athletes impractical and unpopular in applied settings (Taylor et al., 2012; McGuigan et al., 2021). As such, there is a need for exploring simple tools and measures that are readily available to athletes and their coaches (Saw et al., 2018).

To increase our current understanding of acclimatization and responses to training at low altitude among elite endurance athletes, the primary aim of the present study was to monitor the daily variations and time course of changes in selected subjective and objective variables during an altitude training camp at 1,800 m in a group of elite XC skiers and biathletes.

## Methods

### Participants

Thirty-eight members of the Swedish senior national teams in XC skiing (*n* = 26) and biathlon (*n* = 12) were invited to participate in the study. Of these 38, three athletes were excluded because they did not attend the altitude training camp due to injury or illness in the weeks leading up to departure. Three further athletes were excluded due to illness during, and therefore an early departure from, the altitude training camp. This resulted in a final sample of 21 XC skiers (9 women and 12 men) and 11 biathletes (7 women and 4 men), of which nine had won senior Olympic and/or World Championship medals. Participant characteristics according to sport and sex are displayed in Table 1. All athletes were residents at near sea level (i.e., 0–500 m) (Bärtsch et al., 2008) and none had been exposed to altitudes > 1,500 m for more than 7 days in the 6 months prior to the altitude training camp. The study was preapproved by the Regional Ethical Review Board in Umeå, Sweden (ref: 2018-46-31M), and all participants were fully informed about the nature of the study before providing written consent to participate.

**Table 1:** Mean and standard deviation (SD) descriptive characteristics of the participating cross-country skiers and biathletes.

	**Cross-country skiers**				**Biathletes**			
	**Women (*****n*** **= 9)**		**Men (*****n*** **= 12)**		**Women (*****n*** **= 4)**		**Men (*****n*** **= 7)**	
	**Mean**	**SD**	**Mean**	**SD**	**Mean**	**SD**	**Mean**	**SD**
Age (y)	25.9	3.3	25.8	2.4	24.2	3.1	27.3	2.8
Body mass (kg)	61.6	4.5	80.9^*^	6.6	64.6	6.8	75.5^*^	5.9
Height (cm)	166.5	4.2	182.8^*^	7.4	169.6	9.0	180.4^*^	4.2
BMI (kg·m^−2^)	22.2	1.0	24.2^*^	0.8	22.4	1.1	23.2	1.0
$\dot{\text{V}}$O_2peak_ (l·min^−1^)	4.03	0.16	5.84^*^	0.37	3.88	0.35	5.15^*^^#^	0.41
$\dot{\text{V}}$O_2peak_ (ml·kg^−1^·min^−1^)	65.7	4.7	72.5^*^	4.9	60.2^#^	3.4	68.2^*^	2.9

* *Significantly different from the women within the same sport (p < 0.05)*.

# *Significantly different from the cross-country skiers of the same sex (p < 0.05)*.

*BMI, body mass index; $\dot{\text{V}}$O_2peak_, peak oxygen uptake*.

### Study Overview

**Figure 1:**
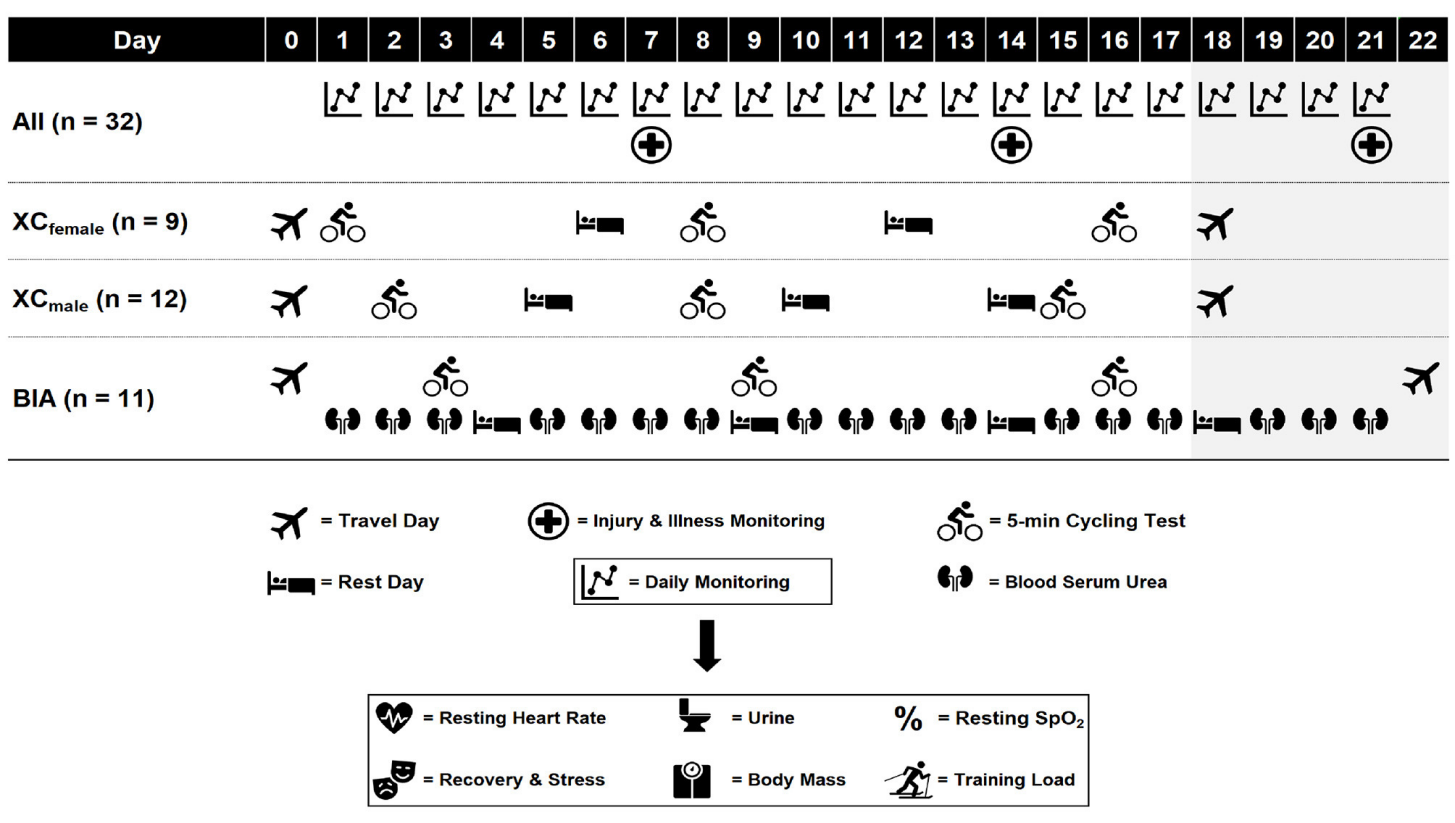
Schematic overview of measures during the altitude training camp. All, all athletes; XC_female_, female cross-country skiers; XC_male_, male cross-country skiers; BIA, biathletes; SpO_2_, peripheral oxygen saturation. Shaded area indicates biathletes only.

The athletes were monitored daily during a pre-season altitude training camp at 1,800 m in August and September 2019. The duration of the training camp was 17 days (18 nights) and 21 days (22 nights), and the hypoxic doses [defined as the elevation of exposure in km multiplied by the duration of exposure in hours (Garvican-Lewis et al., 1985)] were 747 and 890 km·h for the XC skiers and biathletes, respectively. A schematic overview of measures performed at altitude is displayed in Figure 1. Daily monitoring included resting peripheral oxygen saturation (SpO_2rest_), resting heart rate (HR_rest_), body mass (BM), urine specific gravity (USG), urine color (UC), perceived recovery (Rec) and stress, and TL for all athletes, and blood urea nitrogen concentration ([U]) on the morning of all training days for the biathletes only. Due to the larger number of athletes and more complicated logistics, the XC ski coaches chose not to prioritize measurements of [U] in their team. All athletes also registered their injuries and/or illnesses weekly during, and after the first week following the training camp using the Oslo Sports Trauma Research Center questionnaire on health problems (Clarsen et al., 2014).

The female and male XC skiers followed separate training plans set by the coaching staff for each respective sex, while the female and male biathletes followed the same training plan set by the team's head coach (individual adjustments to the training plans were implemented where necessary). All training sessions were performed at 1,200–2,250 m, were designed and led by the national team coaches [employing the *live high-train high* method (Millet et al., 2010)] and were not manipulated or directly influenced as part of the study. Iron status was assessed 4–6 weeks prior to departure to altitude in all athletes and oral iron supplementation was administered to individual athletes by the teams' medical doctors where necessary. In addition to the daily monitoring at altitude, athletes performed a standardized 5-min submaximal cycling test at near sea level (baseline) and this test was repeated at three time points at altitude (Figure 1). Before and after the camp, at near sea level, body composition was assessed in all athletes and the biathletes also performed a standardized roller-skiing performance test.

### Procedures

#### Daily Monitoring

Upon awakening, athletes registered their HR_rest_ using personal HR monitors (Garmin International Inc., Olathe, United States; Suunto, Vantaa, Finland; Polar Electro Oy, Kempele, Finland). They also collected a first morning mid-stream urine sample for the assessment of UC, which was determined by the athletes using a standardized UC scale (Armstrong et al., 1994). After samples were naturally normalized to room temperature (~ 20°C), USG was measured using a digital handheld refractometer (Atago PAL-10s, Atago, Tokyo, Japan). Before breakfast the athletes reported to a field laboratory in a fasted state between ~ 6.30 and 9.00 a.m. for the measurement of SpO_2rest_, BM, Rec, stress and [U], which took a maximum of 3 min to record. Body mass was measured to the nearest 0.1 kg using digital scales (Beuer SR BF 1, Beuer GmbH, Ulm, Germany), SpO_2rest_ was measured using a finger-clip pulse oximeter (Onyx Vantage, Nonin Medical B.V., Netherlands) and perceived Rec and stress were assessed using the overall recovery and stress items from the Short Recovery and Stress Scale (Kellmann and Kölling, 2019). [U] was measured in the biathletes from finger-prick capillary blood samples and analyzed using the Reflotron Plus system (Roche Diagnostics Scandinavia AB, Solna, Sweden). The Reflotron Plus was calibrated according to the manufacturer's instructions on each morning of testing, and the same researcher collected all blood samples. Training load (arbitrary units: arb. units) was quantified as total training duration (min) x session rating of perceived exertion (sRPE) rated on a modified Borg CR10 scale (Foster et al., 2001). Each athlete's sRPE was collected within 10 min of completing each training session. Training duration for each training session was retrieved from the athletes' training diaries and cross-checked against data retrieved from each athlete's personal HR monitor.

#### Submaximal Cycling Test

The 5-min submaximal cycling tests were performed on stationary cycle ergometers (XC skiers: Ergomedic 828e, Monark Exercise AB, Vansbro, Sweden; biathletes: Monark LC, Monark Exercise AB, Vansbro, Sweden) at a constant work rate of 120 W for the women and 160 W for the men. The short 5-min duration and relatively low work rates were chosen to ensure that the submaximal exercise remained moderate at altitude, particularly during the initial days of the camp. In addition, logistical restraints meant that the test needed to be easily included as a warm-up to a subsequent training session, and have minimal impact on the athletes' planned training and recovery. Exercising SpO_2_ (SpO_2work_) and HR (HR_work_) measures were recorded during the final 30 s of the test using a finger-clip pulse oximeter. Immediately after completion of the test, athletes rated their perceived exertion [RPE_work_; 6–20 Borg scale (Borg, 1970)] and blood lactate concentration ([La^−^]_work_) was measured from finger-prick capillary blood samples (XC skiers: Lactate Scout+, EKF Diagnostics GmbH, Barleben, Germany; biathletes: Lactate Pro, Arkray KDK, Kyoto, Japan).

#### Body Composition

Lean mass, fat mass and total BM were determined using dual X-ray absorptiometry (DXA, GE Lunar iDXA, GE Healthcare, Buckinghamshire, UK) 4 ± 4 days and 4 ± 3 days before the day of departure and after the day of return from the altitude training camp, respectively. Measurements were performed according to best practice recommendations for densitometry in athletes (Hind et al., 2018).

#### Submaximal Roller-Ski Test

The submaximal incremental roller-ski tests were carried out according to Laaksonen et al. (2020). Briefly, the incremental test consisted of a standardized 6-min warm-up followed by 3–5 stages each lasting 4 min and separated by 1-min rest intervals. The women began the submaximal test at 7 km·h^−1^ and an inclination of 3.5°, and the men commenced at 8 km·h^−1^ and 4.5°. The speed was increased by 1.5 km·h^−1^ per stage until the following criteria were met: RER > 1.0, $\dot{\text{V}}$_E_·$\dot{\text{V}}{\text{O}}_{2}^{-1}$ > 30, HR ≥ 90% of known HR_max_ and a RPE ≥ 16 out of 20. The tests were conducted 10 ± 3 days and 7 ± 3 days before the day of departure and after the day of return from the altitude training camp, respectively, to identify changes in speed at a blood lactate concentration of 4 mmol·L^−1^ (Speed_@4mmol_). Blood lactate concentration was measured from finger-prick capillary blood samples (Biosen 5140, EKF diagnostic GmbH, Magdeburg, Germany). Respiratory variables were measured throughout the test using an AMIS 2001 model C ergospirometry system (Innovision A/S, Odense, Denmark). For logistical reasons (i.e., the time taken to test 38 athletes using the same equipment, with the same test personnel and at the same laboratory, which in many cases was far away from their home towns), submaximal roller-ski tests were not completed by the XC skiers.

### Statistical Analyses

Data are expressed as mean ± SD unless stated otherwise. All statistical tests were carried out using R version 3.6.3 (R Core Team, Vienna, Austria, 2020) and the alpha level was set at *p* < 0.05. Data from two athletes who became ill during the camp, but did not return home, were partially removed from the analyses (2 and 3 illness days, respectively, as well as 2 recovery days were removed from each participant's data). Three athletes who had either planned early returns or returned home due to illness after completing > 7 days of the training camp were included in the analyses of day-to-day changes but were removed from the pre-post analyses.

Age, total BM, height, body mass index (BMI) and $\dot{\text{V}}$O_2peak_ were compared between sexes and sports using independent sample *t*-tests. Normality of the data was assessed using the Shapiro-Wilks test of normality (α = 0.05). Two separate linear mixed-effects models were used to assess differences in total TL between the first and second weeks in all the athletes and between the first, second and third weeks in the biathletes only (since only the biathletes completed three full weeks of training). Furthermore, separate linear mixed-effects models, incorporating TL as a fixed effect, were used to assess the time course of changes in SpO_2rest_, HR_rest_, BM, USG, UC, Rec, stress and [U] throughout the camp. Time was initially modeled as a continuous variable to assess the slope of each variable over time, then as a discrete variable, to allow comparisons in the mean change at each time point from day one at altitude. Finally, separate linear mixed-effects models were used to assess changes in SpO_2work_, HR_work_ and [La^−^]_work_ during the 5-min submaximal cycling test. In all models, athlete was included as a random effect with random intercepts and/or slopes. The *lme4* package (Bates et al., 2015) was used to fit all linear mixed-effects models. Model selection relied on Akaike's information criterion and assumptions were checked by visual inspection of residual plots. *Post-hoc* comparisons were performed using the *emmeans* package (Lenth, 2020). Within-subject coefficients of variation (CV; i.e., [SD/mean] × 100%) were established to quantify the individual variation in the daily measures (i.e., in SpO_2rest_, HR_rest_, BM, USG, UC, Rec, stress and [U]) (Schabort et al., 1998). Unbiased estimates of the mean within-subject CV were calculated by taking the square root of the average of the square of the CVs of individual participants (Schabort et al., 1998). Changes in RPE during the 5-min submaximal cycling test were assessed using a Friedman's two-way ANOVA by ranks test, while changes in Speed_@4mmol_ from before to after the camp were assessed using a one-tailed Wilcoxon signed-rank test.

## Results

### Training Load

**Figure 2:**
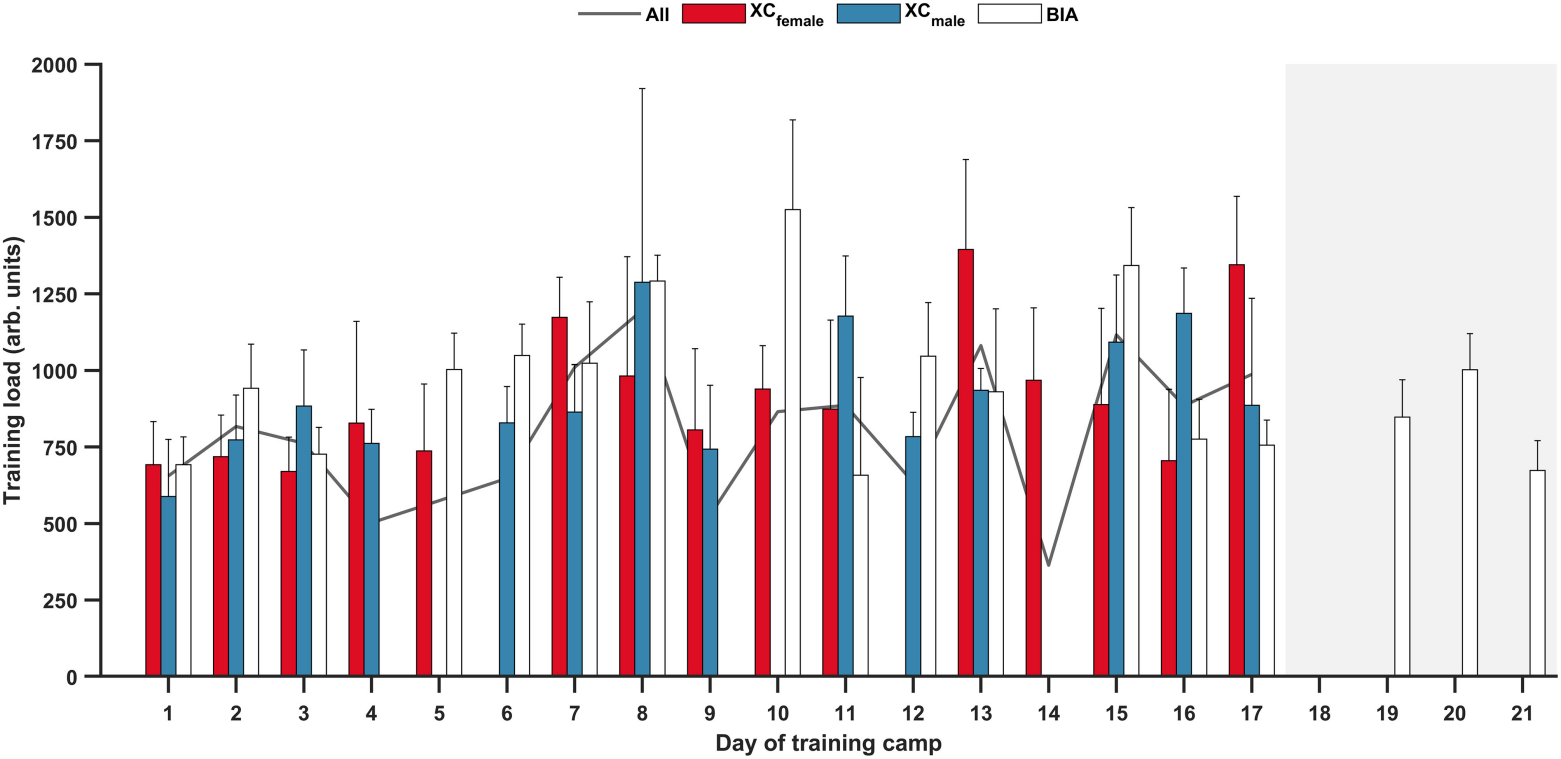
Daily training load (mean ± SD) for female cross-country skiers (XC_female_); male cross-country skiers (XC_male_); biathletes (BIA); and all athletes (All, mean only) during the training camp. Shaded area indicates biathletes only.

Mean daily TL across the training camp was 782 ± 438 arb. units (Figure 2). There was no significant interaction between week and sport for total TL (*F*_(1,21.86)_ = 2.939, *p* = 0.101), nor was there a significant main effect of sport for total TL (*F*_(1,22.40)_ = 3.426, *p* = 0.077). The main effect for week, however, showed that total TL was significantly higher during the second week (5,580 ± 844 arb. units) compared to the first week (5,019 ± 521 arb. units, *F*_(1,21.86)_ = 7.013, *p* = 0.015). When analyzing the biathletes only there was no difference in total TL between the 3 weeks (week 1: 5,452 ± 275 arb. units; week 2: 5,620 ± 635 arb. units; week 3: 5,396 ± 364 arb. units; *F*_(2,18.19)_ = 0.764, *p* = 0.480).

### Daily Monitoring

**Figure 3:**
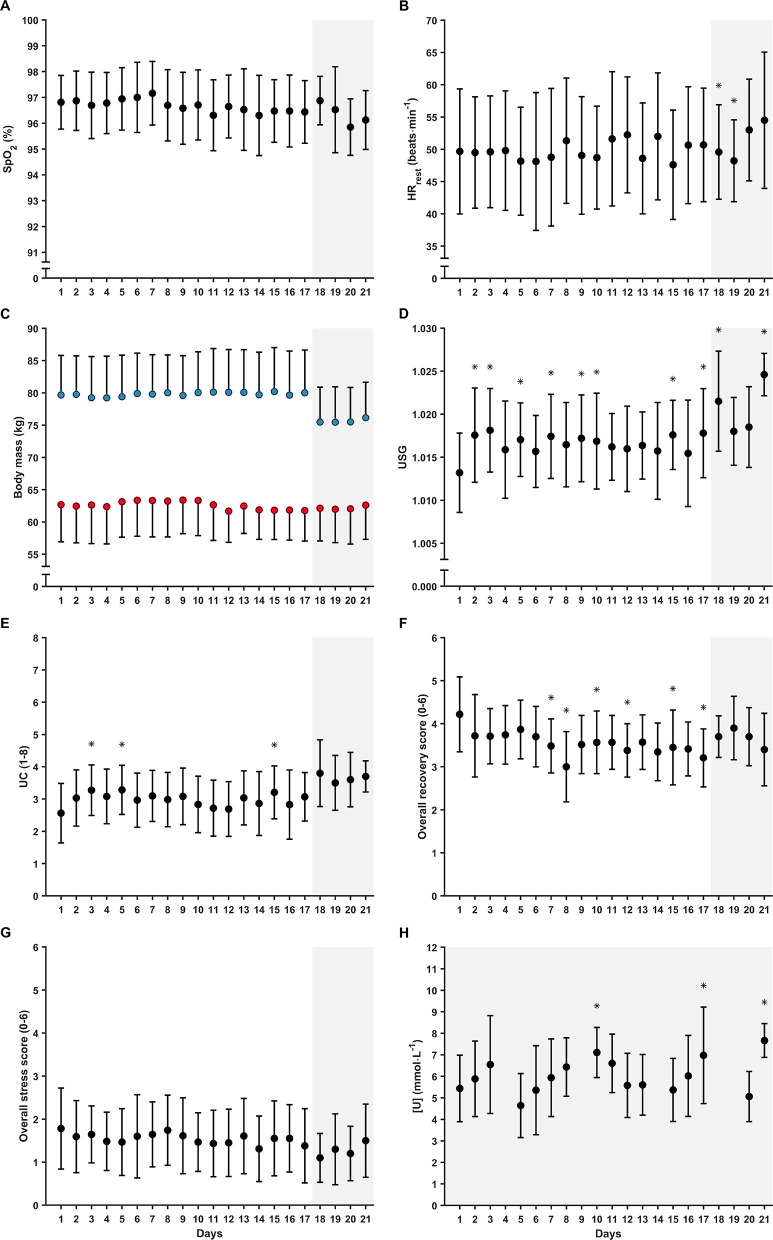
Daily (mean ± SD) **(A)** resting peripheral oxygen saturation (SpO_2rest_); **(B)** resting heart rate (HR_rest_); **(C)** body mass; **(D)** urine specific gravity (USG); **(E)** urine color (UC); **(F)** overall recovery; **(G)** overall stress; and **(H)** blood urea nitrogen concentration ([U], biathletes only) during the training camp. Full circles, all participants; red circles, women; blue circles, men. Shaded areas indicate biathletes only. *Significant effect of time modeled as a discrete variable (i.e., in relation to day 1 of the camp; *p* < 0.05).

Daily measures during the training camp are displayed in Figure 3 for SpO_2rest_, HR_rest_, BM, USG, UC, Rec, stress and [U]. There was a significant effect of time modeled as a continuous variable for BM (b = −0.002, 95% CI [−0.002, 0.004], *F*_(1,30.40)_ = 4.804, *p* = 0.036), Rec (b = −0.023, 95% CI [−0.036, −0.010], *F*_(1,29.67)_ = 12.567, *p* < 0.001), stress (b = −0.016, 95% CI [−0.031, −0.002], *F*_(1,29.29)_ = 4.896, *p* = 0.035) and [U] (b = 0.046, 95% CI [0.017, 0.075], *F*_(1,159.54)_ = 9.775 *p* = 0.002). By contrast, there was no effect of time modeled as a continuous variable for SpO_2rest_ (b = −0.014, 95% CI [−0.030, 0.002], *F*_(1,506.57)_ = 3.043, *p* = 0.082), HR_rest_ (b = −0.076, 95% CI [−0.169, 0.018], *F*_(1,24.94)_ = 2.684, *p* = 0.114), USG (b < −0.001, 95% CI [> −0.001, <0.001], *F*_(1,504.83)_ = 1.9877, *p* = 0.159) or UC (b = 0.006, 95% CI [−0.002, 0.001], *F*_(1,29.80)_ = 0.634, *p* = 0.432).

There was a significant effect of time modeled as a discrete variable for HR_rest_ (*F*_(20,315.57)_ = 1.929, *p* = 0.010), BM (*F*_(20,345.85)_ = 2.418, *p* < 0.001), USG (*F*_(20,478.73)_ = 3.463, *p* < 0.001), UC (*F*_(20,482.89)_ = 2.803, *p* < 0.001), Rec (*F*_(20,364.86)_ = 2.770, *p* < 0.001) and [U] (*F*_(17,143.13)_ = 6.884, *p* < 0.001). By contrast, there was no effect of time modeled as a discrete variable for SpO_2rest_ (*F*_(20,482.83)_ = 1.167, *p* = 0.278) or stress (*F*_(20,365.37)_ = 1.407, *p* = 0.115). Mean within-subject CVs for the daily measures ranged from 0.4% to 69.0% (Table 2).

**Table 2:** Mean and standard deviation (SD) within-subject coefficient of variation (CV, in %) for the daily measures.

	**Mean**	**SD**
SpO_2rest_	1.0	0.8
HR_rest_	7.2	5.1
Body mass	0.8	0.5
USG	0.4	0.3
Urine color	23.7	18.3
Overall recovery	17.9	13.4
Overall stress	69.0	118.8
[*U*]	21.1	14.3

*HR_rest_, resting heart rate; SpO_2rest_, resting oxygen saturation; [U], blood urea nitrogen concentration; USG, urine specific gravity*.

### Submaximal Cycling Test

**Figure 4:**
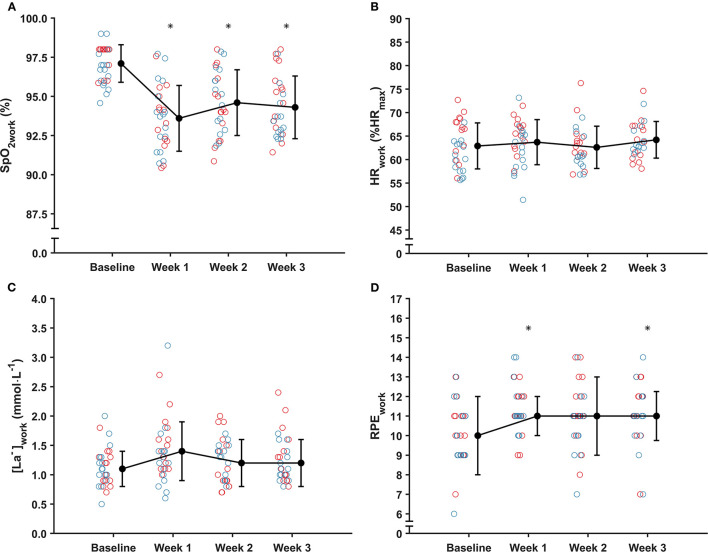
Mean ± SD **(A)** arterial oxygen saturation (SpO_2work_); **(B)** relative heart rate (HR_work_); **(C)** blood lactate concentration [La^−^]_work_; and median ± IQR **(D)** rating of perceived exertion (RPE_work_) during the 5-min submaximal cycling test at baseline (i.e., near sea level) and on three weekly occasions during the training camp (i.e., at 1,800 m). Red circles, women; blue circles, men. *Significantly different from baseline (*p* < 0.05).

The SpO_2work_, HR_work_, [La^−^]_work_ and RPE_work_ measured at baseline and during the three weekly tests at altitude are displayed in Figure 4. There was a significant effect of time on SpO_2work_ (*F*_(3,86.81)_ = 36.1, *p* < 0.001). *Post-hoc* comparisons revealed that SpO_2work_ was lower at all time points at altitude compared to baseline, with mean differences of −3.5% (95% CI [−4.5, −2.5], *t*_(86.30)_ = 9.793, *p* < 0.001), −2,6% (95% CI [−3.5, −1.6], *t*_(86.80)_ = 7.108, *p* < 0.001) and −2.8% (95% CI [−3.8,−1.8], *t*_(87.10)_ = 7.624, *p* < 0.001) at weeks 1, 2 and 3, respectively. However, there were no differences between any of the time points at altitude (all *p* > 0.05). There was a significant effect of time on RPE_work_ ($\chi_{\left(3\right)}^{2}$ = 14.459, *p* = 0.002) and *post-hoc* comparisons showed significant differences from baseline (median [range], 10 [6–13]) to week 1 (11 [9–14], *p* = 0.037) and week 3 (11 [7–14], *p* = 0.010), but not from baseline to week 2 (11 [7–14], *p* = 0.104) or between any of the time points at altitude (all *p* > 0.05). There was no effect of time on HR_work_ (*F*_(3,79.62)_ = 2.0, *p* = 0.123) or [La^−^]_work_ (*F*_(3,82.45)_ = 2.048, *p* = 0.114).

### Illness

In total, 15 illness episodes were reported by 15 different athletes (8 XC skiers, 7 biathletes). Ten episodes were reported during the altitude training camp, of which four resulted in the athlete returning home from the camp early (3 XC skiers, 1 biathlete), while the other five episodes were reported in the first week after the camp (2 XC skiers, 3 biathletes). Eleven out of the 15 illness episodes were reported ≤ 4 days after the outbound or homebound flight. Median [range] training time lost was 2 [0–9] days·episode^−1^ and the most common symptoms reported were indicative of respiratory tract infections (i.e., blocked or runny nose, fever, sneezing, sore throat; 8 episodes) or gastrointestinal infections (i.e., abdominal pain, diarrhea, nausea; 6 episodes). One episode had unspecific symptoms (i.e., swollen glands, fatigue/malaise).

### Body Composition

**Figure 5:**
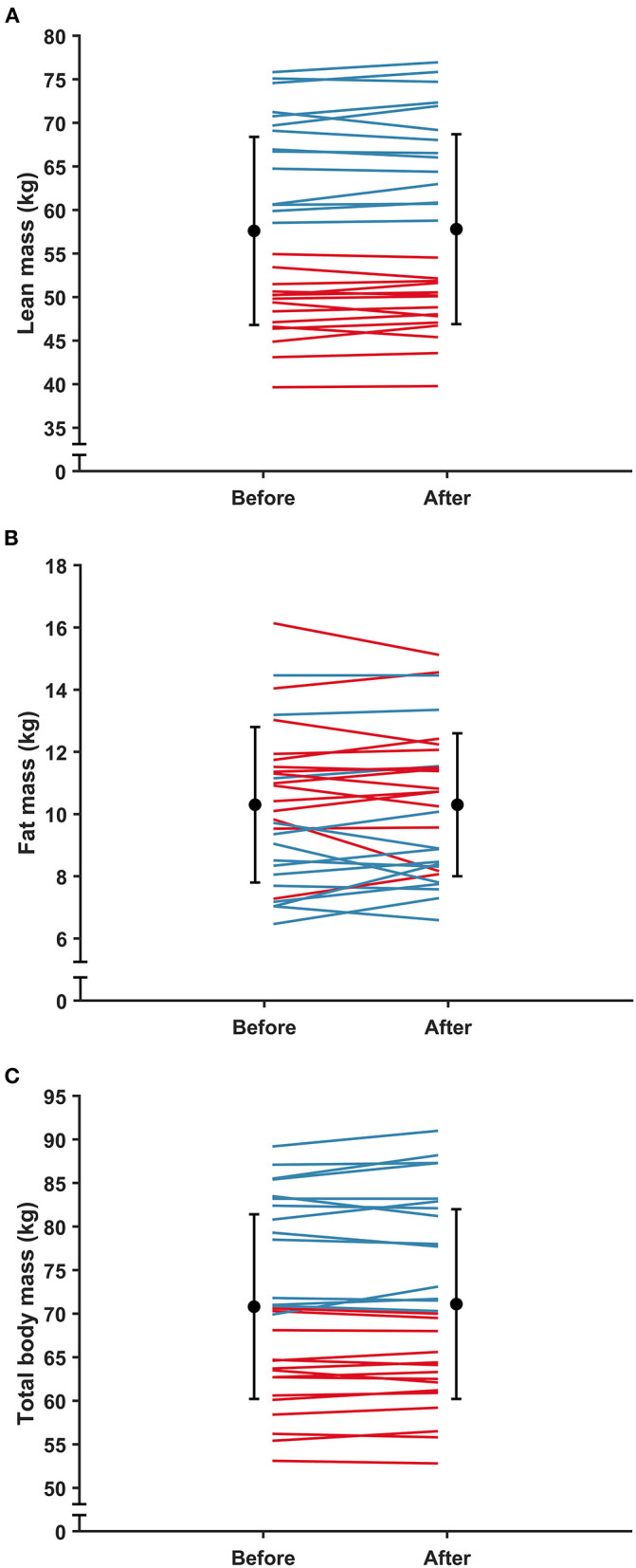
Mean ± SD **(A)** lean mass; **(B)** fat mass; and **(C)** total body mass before and after the training camp. Red lines, women; blue lines, men.

There were no significant interactions between time and sport for lean mass (*F*_(1,27.00)_ = 0.190, *p* = 0.667), fat mass (*F*_(1,27.00)_ = 1.473, *p* = 0.235) or total BM (*F*_(1,27.00)_ = 0.114, *p* = 0.295). Main effects of time revealed no significant differences in lean mass (b = 0.3, 95% CI [−0.2, 0.8], *F*_(1,27.00)_ = 0.860, *p* = 0.362), fat mass (b = 0.2, 95% CI [−0.2,0.5], *F*_(1,27.00)_ = 0.010, *p* = 0.923) or total BM (b = 0.1, 95% CI [−0.1, 1.0], *F*_(1,27.00)_ = 0.677, *p* = 0.418) from before to after the camp (Figure 5). Similarly, main effects of sport revealed no significant differences in lean mass (b = −4.9, 95% CI [−13.2, 3.3], *F*_(1,27.00)_ = 1.428, *p* = 0.242), fat mass (b = 1.2, 95% CI [−0.6, 3.0], *F*_(1,27.00)_ = 1.205, *p* = 0.282) or total BM (b = −4.0, 95% CI [−12.2, 4.2], *F*_(1,27.00)_ = 1.042, *p* = 0.316) between the XC skiers and biathletes.

### Submaximal Roller-Ski Test

Speed_@4mmol_ did not increase among the biathletes from before to after the camp (median [range], 2.3% [−8.5–5.3], *p* = 0.410). However, removing the four biathletes who became ill during or immediately after the camp from the analysis revealed a significant increase in Speed_@4mmol_ for the remaining five athletes (4.2% [2.2–5.3], *p* = 0.031).

## Discussion

The primary purpose of this study was to describe the daily variations and time course of changes in selected subjective and objective variables during an altitude training camp at 1,800 m in senior national team XC skiers and biathletes. The main findings showed that: (1) SpO_2rest_ and HR_rest_ did not change systematically throughout the altitude training camp; (2) body composition (i.e., lean and fat mass) and total BM did not change from before to after the camp; (3) 11 out of 15 illness episodes were reported within four days of the outbound or homebound flight; and (4) the biathletes who remained free of illness improved their Speed_@4mmol_ from before to after the camp by ~ 4%.

### Peripheral Oxygen Saturation

The measurement of SpO_2rest_ has been suggested as a useful tool for monitoring athletes' responses to altitude, as a gradual increase in SpO_2rest_ over several days or weeks after arrival at altitude can be an indicator of healthy hypoxic adaptation (Mujika et al., 2019). In addition, the level of desaturation at rest has been suggested as an indicator of sensitivity to hypoxia and could, therefore, potentially be used to individualize training at altitude (Mujika et al., 2019). However, SpO_2rest_ did not change significantly throughout the altitude training camp in the present study (Figure 3A). This finding was unexpected, particularly given that the skiers in the present study live at low altitudes and experience relatively few days of altitude training each year (typically 1–2 training camps per year each lasting 2–3 weeks). A faster acclimatization phase or attenuated acclimatization responses might have been expected in athletes with more extensive altitude training experience due to *hypoxic memory* (Mujika et al., 2019). While several studies have reported an initial reduction followed by a gradual increase in SpO_2rest_ during altitude training camps at low to high altitudes (e.g., 1,360–3,500 m) (Brugniaux et al., 2006; Buchheit et al., 2013; Saw et al., 2018), conflicting findings, similar to those of the present study, have also been presented. For example, a group of German national team middle- and long-distance runners showed no systematic change in SpO_2rest_ during a 3-week altitude training camp at ~ 2,100 m (Sperlich et al., 2016). This inconsistency in findings could be explained by differences in the time interval and/or timing of the SpO_2rest_ measures [i.e., nocturnal (Brugniaux et al., 2006; Chapman et al., 2016) vs. after awakening (Buchheit et al., 2013; Sperlich et al., 2016; Saw et al., 2018)], as SpO_2rest_ can be sensitive to the periodic breathing that occurs at altitude and the circadian rhythm (Tannheimer and Lechner, 2019), and/or by differences in the living altitudes. Also, the unchanged SpO_2rest_ observed at the group level in the present study does not reflect the different individual responses during the 3-week altitude training camp. Furthermore, the altitude may not have been high enough to demonstrate the expected pattern described above (Millet et al., 2010). Hence, based on the available evidence, further research is needed to determine the usefulness of SpO_2rest_ as a measure of hypoxic adaptation, also at an individual level, in elite endurance athletes at the low to moderate altitudes (i.e., ~ 1,400–2,500 m) typically encountered by this group during training and competitions.

An alternative to the relatively short but practical 1-min SpO_2rest_ measurement period used in the present study would be to use nocturnal measurements (Brugniaux et al., 2006; Chapman et al., 2016). Previously, such measurements required expensive, specialist equipment (Brugniaux et al., 2006; Chapman et al., 2016), which made daily collection with a large group of elite athletes impractical. However, with recent advances in consumer HR-monitor technology, and with manufacturers now offering wrist-worn pulse oximetry in their devices, both on-the-spot and overnight measurements may be more feasible in elite sports settings in the future (Hermand et al., 2021).

### Resting Heart Rate

HR_rest_ did not change systematically throughout the altitude training camp (Figure 3B). This observation is consistent with results reported by Saw et al. (2018), where changes in HR_rest_ over time at altitude (4–19 days at 1,360–2,320 m) were unclear in French and Spanish national team swimmers and members of a UCI World Tour cycling team. In contrast, Sperlich et al. (2016) reported that HR_rest_ decreased from day one to the end of a 21-day training camp at ~ 2,100 m in the aforementioned German middle- and long-distance runners. Similarly, but at a considerably higher altitude (~ 3,600 m), Buchheit et al. (2013) reported an increase in HR_rest_ in the first week, followed by normalization to baseline values in the second week of a 15-day training camp in a group of young (~ 16 y) male soccer players. An initial increase followed by a gradual decrease in HR_rest_ has been regarded as a typical response to sojourns at altitude (Bärtsch and Saltin, 2008; Saw et al., 2018). Assuming that the increase in HR_rest_ primarily occurs to accommodate the reduction in SpO_2_ (Saw et al., 2018), the lack of change in SpO_2rest_ in the present study might explain the lack of change in HR_rest_. It might also be speculated that a greater hypoxic dose (i.e., living/training at higher altitudes) would be necessary to induce changes in HR_rest_ in elite endurance athletes, due to their already well-developed stroke volume and vagal tone (D'Andrea et al., 2002). However, interpretation of repeated HR_rest_ measures over time is complex, since changes in plasma volume, autonomic activity and oxygen partial pressure may all influence HR (Buchheit, 2014).

Another HR-derived measure that could have provided additional objective information regarding physiological adaptation and fatigue state is heart rate variability (HRV). Indeed, HRV has been shown to be useful in monitoring adaptation and fatigue states during altitude training in XC skiers, biathletes and Nordic combined athletes (Schmitt et al., 2008, 2018). However, HRV responses are highly variable both within and between individuals, hence long-term monitoring of HRV indices is necessary to understand each athlete's unique HRV fingerprint and make useful inferences (Plews et al., 2013, 2017). As the teams did not use HRV systematically in their regular training it was not included in the present study. Taken together, considering the inconsistent results in the current literature, more research is warranted to determine the usefulness of HR_rest_ and possibly even HRV as tools for monitoring responses to altitude in elite endurance athletes at relevant altitudes for these individuals.

### Urine Specific Gravity and Urine Color

Increased respiratory water loss and diuresis as early responses to altitude exposure typically lead to increased fluid losses at rest and during training at altitude, potentially putting athletes at an increased risk for dehydration (Stellingwerff et al., 2019). Hence, it is recommended to monitor urinary indices, in combination with BM changes, to continuously assess the hydration status of athletes at altitude (Mujika et al., 2019; Stellingwerff et al., 2019). In the present study, USG and UC obtained from first morning urine samples did not systematically change throughout the altitude training camp, although the measures were significantly higher on certain days compared to the first day of the camp (Figures 3D,E). Despite these findings, mean USG remained below the recommended threshold of 1.020, above which would be indicative of dehydration (Sawka et al., 2007), on all days except for day 18 (1.022 ± 0.006) and day 21 (1.025 ± 0.003). When also considering the maintenance of BM, the present results suggest that the group generally remained hydrated throughout the camp. It is worth highlighting that USG, UC, and BM results were communicated daily to the athletes and their coaches from health and performance perspectives, which might have attenuated the potential adverse effects of altitude on hydration status by increasing the awareness of hydration needs. Moreover, it has been suggested that individual TLs and local weather conditions probably influence hydration status to a greater extent than the potentially milder hypoxic effects experienced at low to moderate altitudes (Stellingwerff et al., 2019).

### Urea

Increased energy expenditure might lead to a temporal depletion of carbohydrate stores and subsequent protein breakdown for gluconeogenesis and ATP production (Hartmann and Mester, 2000). Since urea is an end product of protein metabolism, the measurement of [U] has been adopted to provide information on muscular and/or metabolic strain in endurance athletes (Hartmann and Mester, 2000; Meeusen et al., 2013). As such, this is a routine measurement used by the national biathlon team (but not the XC team) recruited to the present study. The results showed a small but significant increase in [U] over time at altitude (Figure 3H), potentially indicating elevated muscular and/or metabolic strain as the training camp progressed. However, the relatively small daily increase of 0.046 mmol·L^−1^·day^−1^ questions the practical implications of such a change (~ 1 mmol·L^−1^ from day 1 to day 21). In addition, [U] remained within the normal range of 1.7–8.3 mmol·L^−1^ (Hartmann and Mester, 2000) for 157 of the 172 samples in total. So, while there may be a link between accumulated TL and [U] (Hartmann and Mester, 2000), the measurements obtained in the present study imply limited utility of [U] for monitoring acclimatization and training responses in elite endurance athletes at low altitudes.

### Submaximal Cycling Test

The submaximal cycling test showed that SpO_2work_ was significantly reduced at altitude compared to the near-sea-level baseline by 2.6–3.5% at all time points (Figure 4A), while RPE_work_ was significantly increased by one unit at the first and third time points at altitude compared to near sea level (Figure 4D). In contrast, there were no significant changes over time in HR_work_ or [La^−^]_work_ (Figures 4B,C). Similar to the present findings, previous studies on untrained individuals at high altitudes (> 3,000 m) have reported a decrease in SpO_2work_ during submaximal exercise at altitude compared to near sea level or low altitude (<600 m) baselines (Wolfel et al., 1991; Burtscher et al., 2018b). Contrary to the present study, however, these studies also showed concomitant increases in submaximal HR and [La^−^]_work_ at altitude (Wolfel et al., 1991; Burtscher et al., 2018b). An increase in HR during submaximal exercise would be expected, to compensate for the reduced oxygen content of the blood (Fulco et al., 1998). However, it might be speculated that the work rates used in the present study (120 W and 160 W; ~ 65% HR_max_) were too low to induce significant perturbations in HR_work_ and [La^−^]_work_, especially when considering the relatively low altitude and the elite status of the endurance athletes. Further research could focus on developing a standardized exercise protocol that is able to provide reliable and valid measures of sub-maximal exercise tolerance at low altitudes in elite endurance athletes, whilst ensuring that such tests impose minimal interference upon the athletes' priorities of sport-specific training and recovery.

### Illness

In the present study, 11 out of 15 illness episodes were reported within 4 days after the outbound or homebound flight. The reported symptoms were primarily indicative of respiratory tract and gastrointestinal infections (8/15 and 6/15 cases, respectively). Svendsen et al. (2016) have previously reported that international air travel was the single largest risk factor for reporting symptoms of acute infections (i.e., respiratory tract and gastrointestinal infections) in a group of Norwegian national team XC skiers, with athletes approximately five times more likely to report symptoms the following day. Similarly, Schwellnus et al. (2012) reported a two- to three-fold increase in the risk of illness following international air travel to a foreign destination more than five time zones from their home country. Accordingly, the present findings support these previous observations and underline the importance of good traveling routines to minimize the risk of illness in association with international air travel, in general, but specifically in relation to altitude training camps (Wachsmuth et al., 2013; Heikura et al., 2018). Also, the high proportion (~ 45%) of athletes becoming ill during or after this overseas altitude camp raises the question of whether the risk of illness outweighs the potential performance benefits, or whether the use of home-based simulated altitude exposure may be preferable.

### Body Mass and Body Composition

While sojourns at altitudes > 3,000 m can result in significant weight loss (Westerterp and Kayser, 2006; Buchheit et al., 2013), results from studies on athletes residing at low to moderate altitudes that are generally recommended for altitude training (i.e., 1,800–3,000 m), and of similar durations to the present study (~ 3 weeks), have shown either small decreases (Gore et al., 1998) or no change in total BM (Sperlich et al., 2016; Woods et al., 2017; Heikura et al., 2018; Koivisto et al., 2018). Consistent with the studies showing no change (Sperlich et al., 2016; Woods et al., 2017; Heikura et al., 2018; Koivisto et al., 2018), total BM did not change from before to after the camp (Figure 5C). Similarly, lean and fat mass did not change from before to after the camp (Figure 5A,B). In addition, while there was a significant reduction in BM with days at altitude (Figure 3C), the small daily reduction of ~ 2 g·day^−1^ questions the practical implications of such a change (i.e., ~ 40 g from day 1 to day 21). Hence, these results suggest that the athletes were able to maintain energy balance throughout the camp. This is an important practical outcome as failure to maintain BM may, besides leading to other health and performance issues, have attenuating effects on hematological adaptations to hypoxia (McLean et al., 2013; Stellingwerff et al., 2019). The athletes in the present study were catered for by a professional catering company working in consultation with the coaches and practitioners throughout the camp, which may have contributed to this positive outcome.

### Submaximal Roller-Ski Performance

All the biathletes who remained free of illness throughout the altitude training camp and in the week immediately after returning home improved their Speed_@4mmol_ by 2.2–5.3%. This improvement is consistent with Bailey et al. (1998) who reported a 12% increase (from 5.97 ± 0.83 to 6.70 ± 1.13 m·s^−1^) in Speed_@4mmol_ following 4 weeks of endurance training at 1,500–2,000 m in a group of British national team distance runners. In contrast, three out of the four biathletes in the present study who became ill during or in the week immediately after the altitude training camp, but who still completed the camp, showed reductions in their Speed_@4mmol_ by 3.6–8.5%. This result is also consistent with previous research that has reported a negative effect of illness on sea-level competition performance following altitude training in a group of German Olympic swimming team prospects (Wachsmuth et al., 2013). An increase in total Hb_mass_ has been regarded as the primary physiological rationale for applying altitude training to increase subsequent sea-level performance (Stellingwerff et al., 2019), as an increase in Hb_mass_ increases oxygen delivery to exercising muscles. This could potentially explain the increase in submaximal roller-ski performance following the altitude training camp in the present study. While no blood markers could be measured in the present study, 3 weeks of *live high-train high* altitude training at 1,800 m have been shown to be sufficient to significantly increase Hb_mass_ in well-trained runners (Garvican-Lewis et al., 2015), despite 1,800 m generally being regarded as too low to induce an increase in Hb_mass_. Alternatively, the increase in submaximal roller-ski performance may have resulted from non-hematological hypoxia-induced changes, such as an enhanced muscle buffering capacity (Bärtsch and Saltin, 2008; Mujika et al., 2019) or improvements in exercise economy (Millet et al., 2010). Another explanation could be an increase in training quality and recovery during the training camp, where the athletes had the opportunity to eliminate the stresses of everyday life at home (Levine and Stray-Gundersen, 1992). A potential explanation for the reductions in performance could be that illness has an attenuating effect on the Hb_mass_ increase associated with altitude training (McLean et al., 2013; Wachsmuth et al., 2013; Heikura et al., 2018). Alternatively, the diminished performances could simply be a result of a reduction in training load and/or suboptimal training as a result of illness. While acknowledging the low number of athletes, the results from the submaximal roller-ski test suggest that the altitude training camp had a beneficial effect on subsequent submaximal endurance performance at sea level, provided that the athletes remained free of illness. However, with the present study design it cannot be determined whether the effect was due to altitude exposure *per se*, or was a *training camp effect* (Levine and Stray-Gundersen, 1992).

### Strengths and Limitations

As part of two national teams' Olympic-cycle preparations, the present study was designed and required to impose minimal interference upon the athletes' training and daily routines. Also, measuring tools and variables were chosen based on their feasibility in an applied setting. Thus, a major strength is that the study presents a true reflection of the training characteristics and outcomes of an altitude camp with elite endurance athletes using tools that are readily available to athletes and coaches. Whilst care was taken to ensure scientific rigor, some limitations remain. First, no control group was included in the present study. This is unsurprising since all available senior national team XC skiers and biathletes were recruited to the experimental group and no suitable control group exists. Hence, whether the responses were due to the altitude exposure *per se* or were the result of a *training camp effect* cannot be determined (Levine and Stray-Gundersen, 1992). Second, the daily measures collected at altitude were not part of the athletes' regular daily monitoring routines, meaning that baseline and follow-up measures were not available. The athletes were not familiar with the measurement procedures and lacked the required experience and/or equipment to collect the data themselves, making it logistically impossible to collect these data rigorously at different locations around Sweden. Third, ventilatory (e.g., ventilation and hypoxic ventilatory response) and hematological (e.g., hemoglobin mass and concentration) variables were not monitored. Considering that ventilatory responses are especially important in the acute acclimatization and that hematological adaptations are one of the primary long-term adaptations to altitude exposure (Bärtsch and Saltin, 2008), future studies investigating responses to low altitudes in elite endurance athletes could include such measures. In the present study, these measures were not possible due to their invasive and/or time-consuming nature, making their daily use with a large group of elite athletes impractical in an applied setting. Also, athletes and coaches usually lack the required equipment and expertise to carry out such measurements themselves. Fourth, the time point for the submaximal roller-ski test performed after the training camp had to be adapted to the athletes' training schedules. As a result, there was considerable variation in the duration (7 ± 3 days) after the camp that the tests were performed. This may have resulted in the athletes being at different stages in the deacclimatization process when performing the tests, which could have influenced their performance (Chapman et al., 1985). Finally, as mentioned previously, the results of the daily measures were communicated to the athletes and their coaches each day. This is important in real-world scenarios where optimizing the effects of training camps is of utmost importance, but from a scientific perspective may influence athlete behavior.

## Conclusions

The present study was conducted in an ecologically valid environment with elite endurance athletes at an altitude relevant for this group, while using simple tools readily available to the athletes and their coaches. The results showed that measures typically recommended to monitor acclimatization and responses to altitude in athletes, such as SpO_2rest_ and HR_rest_, did not follow the patterns suggested in the literature (e.g., an increase in SpO_2rest_ and a decrease in HR_rest_ over time) (Saw et al., 2018; Mujika et al., 2019). Therefore, further research is needed to explore the utility of these and other measures that could have strong practical relevance to elite endurance athletes, and to understand responses of these athletes at low altitudes typical of competition environments.

## Data Availability Statement

The datasets presented in this article are not readily available because the data contain information that could compromise research participants' privacy and/or consent. Requests to access the datasets should be directed to oyvind.karlsson@miun.se.

## Ethics Statement

The study was preapproved by the Regional Ethical Review Board in Umeå, Sweden (ref: 2018-46-31M) and all participants were fully informed about the nature of the study before providing written consent to participate.

## Author Contributions

ØK carried out the data collection and analyses. KM contributed to the data collection and interpretation of the results. All authors contributed to the study design and manuscript writing, have read and approved the final version of the manuscript, and agree with the order of presentation of the authors.

## Funding

This work was part-financed by the Mid Sweden University and Östersund City Council financial agreement.

## Conflict of Interest

The authors declare that the research was conducted in the absence of any commercial or financial relationships that could be construed as a potential conflict of interest.

## Publisher's Note

All claims expressed in this article are solely those of the authors and do not necessarily represent those of their affiliated organizations, or those of the publisher, the editors and the reviewers. Any product that may be evaluated in this article, or claim that may be made by its manufacturer, is not guaranteed or endorsed by the publisher.
